# Association between gut microbiota and pan-dermatological diseases: a bidirectional Mendelian randomization research

**DOI:** 10.3389/fcimb.2024.1327083

**Published:** 2024-03-18

**Authors:** Yingwei Wang, Tao Yao, Yunlu Lin, Hongping Ge, Bixin Huang, Yu Gao, Jianming Wu

**Affiliations:** ^1^ Department of Dermatology, The Second Affiliated Hospital and Yuying Children’s Hospital of Wenzhou Medical University, Wenzhou, China; ^2^ Department of Cardiology, The Second Affiliated Hospital and Yuying Children’s Hospital of Wenzhou Medical University, Wenzhou, China

**Keywords:** dermatological diseases, gut microbiota, mendelian randomization analysis, inference, therapeutics

## Abstract

**Background:**

Gut microbiota has been associated with dermatological problems in earlier observational studies. However, it is unclear whether gut microbiota has a causal function in dermatological diseases.

**Methods:**

Thirteen dermatological diseases were the subject of bidirectional Mendelian randomization (MR) research aimed at identifying potential causal links between gut microbiota and these diseases. Summary statistics for the Genome-Wide Association Study (GWAS) of gut microbiota and dermatological diseases were obtained from public datasets. With the goal of evaluating the causal estimates, five acknowledged MR approaches were utilized along with multiple testing corrections, with inverse variance weighted (IVW) regression serving as the main methodology. Regarding the taxa that were causally linked with dermatological diseases in the forward MR analysis, reverse MR was performed. A series of sensitivity analyses were conducted to test the robustness of the causal estimates.

**Results:**

The combined results of the five MR methods and sensitivity analysis showed 94 suggestive and five significant causal relationships. In particular, the *genus Eubacterium_fissicatena_group* increased the risk of developing psoriasis vulgaris (odds ratio [OR] = 1.32, p_FDR_ = 4.36 × 10^−3^), *family Bacteroidaceae* (OR = 2.25, p_FDR_ = 4.39 × 10^−3^), *genus Allisonella* (OR = 1.42, p_FDR_ = 1.29 × 10^−2^), and *genus Bacteroides* (OR = 2.25, p_FDR_ = 1.29 × 10^−2^) increased the risk of developing acne; and the *genus Intestinibacter* increased the risk of urticaria (OR = 1.30, p_FDR_ = 9.13 × 10^−3^). A reverse MR study revealed insufficient evidence for a significant causal relationship. In addition, there was no discernible horizontal pleiotropy or heterogeneity.

**Conclusion:**

This study provides novel insights into the causality of gut microbiota in dermatological diseases and therapeutic or preventive paradigms for cutaneous conditions.

## Background

The gut microbiota comprises the entire consortium of microorganisms that inhabit the intestinal tract, with the normal adult gut microbiota predominantly localized within the colon and the distal small intestine. Due to its prolific gene-carrying capacity, it is termed the “second human genome” (Pasolli et al., 2019). Age, sex, lifestyle, and environmental variables significantly affect the composition of the gut microbiota (De Filippo et al., 2010; Takagi et al., 2019). The imperative role of the gut microbiota in upholding intestinal well-being is beyond contention; however, it concurrently preserves the dynamic equilibrium of overall organismal metabolism and immunity by actively participating in a multitude of intricate physiological and biochemical processes.

The skin is one of the distant organs that has been specifically linked to gut microbiota. It has been postulated that signaling pathways governing epidermal keratinization, a pivotal factor in maintaining skin barrier integrity, can be subject to the gut microbiota (Abhishek and Krishnan, 2016). The advent of the gut–skin axis has highlighted a nexus between the gut microbiota and skin disorders, including atopic dermatitis (AD), acne, psoriasis, rosacea, and melanoma (Searle et al., 2020; Fang et al., 2021; Buhaş et al., 2022; Makaranka et al., 2022; Sánchez-Pellicer et al., 2022; Maronese et al., 2023). For instance, in the context of AD, discernible alterations include diminished gut microbial diversity and marked reductions in beneficial microbes, such as *Lactobacillus* and *Bifidobacterium*, juxtaposed with elevated proportions of *Escherichia coli*, *Clostridium difficile*, and *Staphylococcus aureus* (Fang et al., 2021). Additionally, a comprehensive appraisal of psoriasis and gut microbiota has underscored an evident dysbiosis in patients with psoriasis, marked by the depletion of *Lachnospira*, *Faecalibacterium*, and *Akkermansia muciniphila*, while witnessing escalated levels of *E. coli* and *Ruminococcus* (Buhaş et al., 2022). Furthermore, burgeoning research on oral probiotics, prebiotics, and dietary modifications has garnered empirical validation for their potential to ameliorate diverse skin conditions (Ellis et al., 2019; Yu et al., 2020; Sinha et al., 2021). Although previous studies have described interactions between gut microbiota and dermatological diseases, the exact causal relationship remains unclear. Confounding elements such as reverse causation and variables encompassing infections, dietary habits, antibiotic usage, and deleterious lifestyle practices inherent within observational studies may cast a shadow on the conclusion.

Mendelian randomization (MR) analysis, a genetic statistical approach, is based on by Mendel’s second law. It harnesses genetic variants closely entwined with exposure factors as instrumental variables (IVs) to evaluate the statistical causality underpinning exposures and outcomes meticulously. This circumvents the vulnerabilities of conventional statistical methods that are susceptible to extraneous influences, thereby furnishing more robust conclusions (Burgess et al., 2015). Notably, the precedence of the genotype over the phenotype empowers the results to ignore the interference of reverse causality. Two-sample Mendelian randomization (2SMR) utilizes MR methods to estimate the causal estimates of GWAS summary datasets of two distinct studies. In an era where the expansive tapestry of contemporary GWASs explores the genetic variant-disease nexus, the application of 2SMR has emerged as a potent tool for plumbing the depths of causal interrelationships between gut microbiota and skin diseases.

Hence, we employed 2SMR to probe the causal relationship between the gut microbiota and various dermatological diseases, including AD, vitiligo, acne, rosacea, urticaria, seborrheic dermatitis, psoriasis, psoriasis vulgaris, psoriatic arthritis, malignant melanoma, non-malignant melanoma skin cancer, facial aging, and hidradenitis suppurativa. Based on these findings, our aim was to elucidate the involvement of the gut microbiota in dermatological diseases, seeking to provide novel insights into their pathogenesis. This exploration is anticipated to contribute to the development of innovative therapeutic approaches, including but not limited to probiotic therapy, prebiotic therapy, dietary modifications, and fecal microbiota transplantation, thus fostering advancements in dermatological care.

## Methods

**Figure 1:**
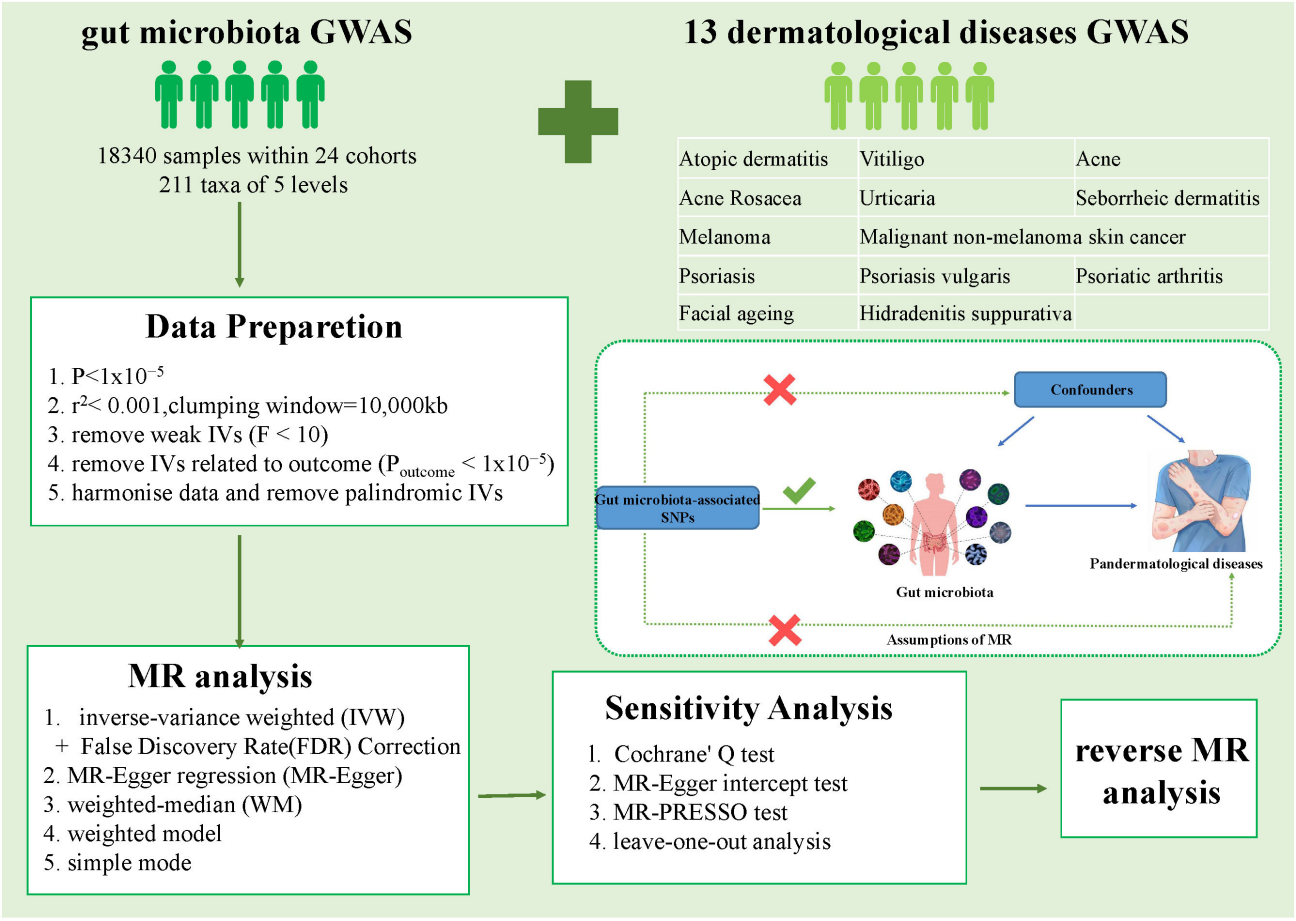
Overall workflow of the study and the premises of Mendelian randomization. GWAS, Genome-Wide Association Study; IVs, instrumental variables; SNP, single nucleotide polymorphisms.

A schematic representation of the analysis is shown in **Figure 1**. The causal relationship between genetically predicted gut microbiota and 13 dermatological diseases was explored using rigorously screened single-nucleotide polymorphisms (SNPs) as IVs for 2SMR and sensitivity analysis. The SNPs were selected to rigorously satisfy three major premises of the MR analysis: First, the relevance premise: IVs are highly linked with the relevant exposure; second, the independence premise: IVs lack any confounders related to the exposure or outcome; and third, the exclusion premise: IVs only influence the outcome via exposure (Davey Smith and Hemani, 2014). Furthermore, reverse MR analysis was performed to evaluate reverse causation. All statistical analysis were performed in the R Version 4.3.1 using packages “TwoSampleMR” (version 0.5.6) and “MR-PRESSO” (version 1.0).

### Data sources

The MiBioGen Consortium provided a GWAS dataset of the human gut microbiota (Kurilshikov et al., 2021). The present investigation integrated profiles of 16S rRNA gene sequencing and genotyping data derived from a cohort comprising 18,340 participants (13,266 participants from Europe), originating from 24 multiethnic population-based cohorts. This encompassed the analysis of 211 distinct bacterial taxa, of which 15 microbial taxa without specific species designations were omitted. Consequently, a comprehensive total of 196 bacterial taxa (consisting of 119 genera, 32 families, 20 orders, 16 classes, and nine phyla) were included within the scope of this study. Dermatological statistics from the most recent large GWAS datasets are available. More specifically, the Early Genetics and Life Course Epidemiology (EAGLE) Consortium offered a GWAS for AD (n = 116,863), which was conducted in 22 cohorts of European ancestry and four cohorts of non-European ancestry. We then obtained the summarized statistics for a GWAS meta-analysis of vitiligo (n = 44,266) conducted among individuals of European ancestry from the GWAS Catalog (GCST004785). GWAS summary data for rosacea (n = 299,421) were derived from the findings of the GWAS on FinnGen R7, while GWAS summary data for acne (n = 44,266), psoriasis (n = 339,050), psoriasis vulgaris (n = 335,993), psoriatic arthritis (n = 333,887), urticaria (n = 340,278), seborrheic dermatitis (n = 309,188), and hidradenitis suppurativa (n = 329,547) were derived from the results of the GWAS on FinnGen R8. The detailed data are available on the FinnGen webpage. In addition, we obtained GWAS data from the UK Biobank for melanoma (n = 375,767) and malignant non-melanoma skin cancer (n = 395,710). Summary statistics for facial aging (n = 423,999) were assessed by the Medical Research Council-Integrative Epidemiology Unit (MRC-IEU). The details of the GWAS information are presented in **Supplementray Table 1**.

### Instrumental variable selection

The subsequent quality assurance processes employed for the section on genetic predictors related to microbiota were utilized to guarantee the correctness of the conclusion regarding the causal effect of gut microbiota on dermatological diseases. SNPs associated with taxa at the locus-wide significance threshold (p <1.0 × 10^−5^) were selected as potential IVs. Subsequently, we meticulously curated independent IVs for each distinct bacterial taxon by conducting linkage disequilibrium analysis (R2 <0.001, window size = 10,000 kb), thereby preemptively curbing the potential for skewed causal estimations (1000 Genomes Project Consortium et al., 2012). We subsequently extracted the pertinent data of the designated SNPs from the GWAS outcome data, systematically excluding SNPs with a direct association with the outcomes (p >1.0 × 10^−5^). To guarantee that the allelic impact of SNPs on exposure and their impact on outcome remained identical throughout the harmonization procedure, palindromic SNPs were eliminated. F-statistics were used to measure the strength of the IVs (Burgess et al., 2011). The F-statistic of each IV was computed using the following formula: $\text{F}=\frac{R^{2}\left(N-2\right)}{1-R^{2}},$ where R^2^ is the percentage of exposure variance that can be accounted for by genetic variations and N denotes the size of the entire exposure GWAS sample. Using the formula ${\text{R}}^{2}=\frac{\beta^{2}}{\beta^{2}+Se^{2}*N},$ where β is the beta value and Se is the standard error, the R^2^ of each IV was determined. The candidate IV sets were devoid of inferior IVs, with F-statistics <10.

### MR analysis

Five effective MR methods were utilized in this investigation to check for causal relationships: The inverse variance weighted (IVW) method employs a meta-analysis amalgamating the Wald estimates of SNPs associated with each taxa, yielding a comprehensive estimation of their impact on distinct dermatoses, which can provide unbiased results in the absence of pleiotropy (Bowden et al., 2015); Based on the instrument strength independent of direct effect (InSIDE), MR-Egger produces a regression result that is consistent with IVW if the intercept term is 0, which denotes the absence of horizontal pleiotropy (Burgess and Thompson, 2015); The weighted median facilitates accurate determination of causality even in scenarios where as much as 50% of the IVs prove invalid (Bowden et al., 2016); The weighted mode estimate has been found to have greater power than MR-Egger to detect causal effects, less bias, and lower type I error rates when the InSIDE assumption is violated (Hartwig et al., 2017); While the statistical efficacy may not rival that of the IVW, the simple mode still yields robust outcomes even amidst the presence of pleiotropy (Milne et al., 2017). Given the slightly augmented statistical potency of the IVW method in summary-level MR, it was adopted as the principal outcome, with the remaining four methods serving as a supplementary analysis. The random-effects IVW regression model, if devoid of SNPs that breach the IV assumption of independence, provides an unbiased estimate of the causal impact that endures scrutiny (Burgess et al., 2013). To further safeguard against spurious findings, we implemented the False Discovery Rate (FDR) correction to establish a threshold for multiple testing significance, denoted as P_FDR_ <0.05 (Storey and Tibshirani, 2003). FDR correction was executed, with p_FDR_ <0.05, being deemed indicators of significant causal relationships. Suggestive causalities between gut microbiota and dermatological diseases were discerned at p <0.05, but p_FDR_ ≥0.05 (Gu et al., 2023).

### Sensitivity analysis

Both the IVW and MR-Egger approaches employed Cochran’s Q statistic to assess the presence of heterogeneity, with a significance threshold of 0.05. Cochran’s Q statistic, with a p-value below 0.05, would provide evidence of notable heterogeneity among the IVs (Bowden et al., 2018). Following this, we conducted an iterative leave-one-out analysis, removing individual SNPs to ascertain the singular influence of each SNP (Burgess, 2014). In addition, we examined the MR-Egger intercept to investigate the potential presence of horizontal pleiotropy. We considered the horizontal pleiotropy of IVs to have an insignificant impact on causal inferences if the corresponding p-value exceeded 0.05 (Bowden et al., 2015). In conjunction with this analysis, we employed a more refined version of the MR-PRESSO global test. A p-value exceeding 0.05 in the MR-PRESSO global test indicated the absence of pleiotropy. If evidence of horizontal pleiotropy was detected among the selected single nucleotide polymorphisms (SNPs), the analysis was repeated after excluding these pleiotropic SNPs (Verbanck et al., 2018).

### Bidirectional MR analysis

To probe the potential causal implications of dermatological diseases on the identified bacterial genera, reverse MR analysis was conducted, employing SNPs associated with each dermatological disease. For the selection of IVs, we opted for SNPs falling below the genome-wide statistical significance threshold (5 × 10^−8^), except for rosacea (p <5 × 10^−7^), seborrheic dermatitis (p <5 × 10^−7^), and hidradenitis suppurativa (p <5 × 10^−6^), due to the insufficient number of SNPs attainable for MR analysis under the conventional threshold. Subsequent steps in the analysis closely followed the previously outlined MR procedure. Notably, in instances where only a single SNP is present, we employ the Wald ratio as the analytical method.

## Results

### Selection of instrumental variables

Following linkage disequilibrium clumping, we identified 124, 223, 279, 469, and 1,531 SNPs associated with the gut microbiota at the phylum, class, order, family, and genus levels, respectively, displaying a permissive statistical threshold (p <1 × 10^−5^). After harmonization, retained SNPs were used as IVs in the formal MR analysis. All the IVs used in the identified causal associations are listed in **Supplementray Table 2**. The F-statistic values for these SNPs ranged between 17 and 85, indicating a robust IV selection. Notably, an inherent hierarchical relationship within bacterial taxa classifications occasionally led to substantial SNP overlap, as exemplified by the *family Bacteroidaceae* and the *genus Bacteroides*.

### Significant causal associations between gut microbiota and dermatological diseases

Five MR methods were used to explore the causal relationship between each pair of bacterial taxa and dermatological diseases. We used IVW as the primary outcome indicator. After multiple-testing correction, five significant causal associations (p < 0.05, p_FDR_ < 0.05) associated with the three skin diseases were monitored. We found that the *genus Eubacterium_fissicatena_group* (OR = 1.32, 95% confidence interval [CI] = 1.16–1.50, p = 3.66 × 10^−5^, p_FDR_ = 4.36 × 10^−3^) was causally associated with psoriasis vulgaris; the *family Bacteroidaceae* (OR = 2.25, 95%CI = 1.48–3.42, p = 1.37 × 10^−4^, p_FDR_ = 4.39 × 10^−3^), *genus Allisonella* (OR = 1.42, 95%CI = 1.18–1.70, p = 2.16 × 10^−4^, p_FDR_ = 1.29 × 10^−2^) and *genus Bacteroides* (OR = 2.25, 95%CI = 1.48–3.42, p = 1.37 × 10^−4^, p_FDR_ = 1.29 × 10^−2^) were causally associated with acne; while the *genus Intestinibacter* (OR = 1.30, 95%CI = 1.14–1.48, p = 7.67 × 10^−5^, p_FDR_ = 9.13 × 10^−3^) was causally associated with urticaria (**Table 1**).

**Table 1:** MR estimation of the significant causal relationships and tests for heterogeneity and horizontal pleiotropy.

Exposure	Outcome	Method	nSNP	OR(95%CI)	*p*-value	*p-FDR*	*P*_Heterogeneity_	*P*_Pleiotropy_	*P*_Global Test_
genus_Eubacterium fissicatena group	Psoriasis vulgaris	MR Egger	9	1.093(0.556,2.150)	8.03E-01	4.36E-03	0.628	0.601	0.698
		Weighted median		1.281(1.074,1.528)	5.90E-03				
		Inverse variance weighted		1.316(1.155,1.499)	3.66E-05				
		Simple mode		1.506(1.111,2.041)	2.99E-02				
		Weighted mode		1.149(0.863,1.529)	3.70E-01				
family_Bacteroidaceae	Acne	MR Egger	8	1.643(0.152,17.716)	6.96E-01	4.39E-03	0.339	0.800	0.426
		Weighted median		1.772(1.026,3.060)	4.01E-02				
		Inverse variance weighted		2.253(1.484,3.421)	1.37E-04				
		Simple mode		1.579(0.680,3.666)	3.23E-01				
		Weighted mode		1.537(0.778,3.038)	2.56E-01				
genus_Allisonella	Acne	MR Egger	8	2.212(0.603,8.121)	2.77E-01	1.29E-02	0.323	0.522	0.389
		Weighted median		1.362(1.076,1.726)	1.04E-02				
		Inverse variance weighted		1.416(1.178,1.702)	2.16E-04				
		Simple mode		1.468(1.012,2.130)	8.26E-02				
		Weighted mode		1.405(1.004,1.968)	8.80E-02				
genus_Bacteroides	Acne	MR Egger	8	1.643(0.152,17.716)	6.96E-01	1.29E-02	0.339	0.800	0.420
		Weighted median		1.772(0.994,3.159)	5.25E-02				
		Inverse variance weighted		2.253(1.484,3.421)	1.37E-04				
		Simple mode		1.579(0.707,3.525)	3.02E-01				
		Weighted mode		1.537(0.725,3.260)	2.99E-01				
genus_Intestinibacter	Urticaria	MR Egger	14	1.237(0.824,1.857)	3.24E-01	9.13E-03	0.831	0.801	0.858
		Weighted median		1.225(1.015,1.477)	3.41E-02				
		Inverse variance weighted		1.301(1.142,1.483)	7.67E-05				
		Simple mode		1.237(0.924,1.656)	1.76E-01				
		Weighted mode		1.224(0.952,1.573)	1.38E-01				

nSNP, number of the SNP used as the IVs for the MR analyses;OR, odds ratio; 95% CI, 95% confidence interval; p-FDR, p-value after False Discovery Rate correction; P_Heterogeneity_, p-value of the heterogeneity test; P_Pleiotropy_, p-value of the intercept of the MR Egger; P_Global test_, p-value of the MR-PRESSO global test.

**Figure 2:**
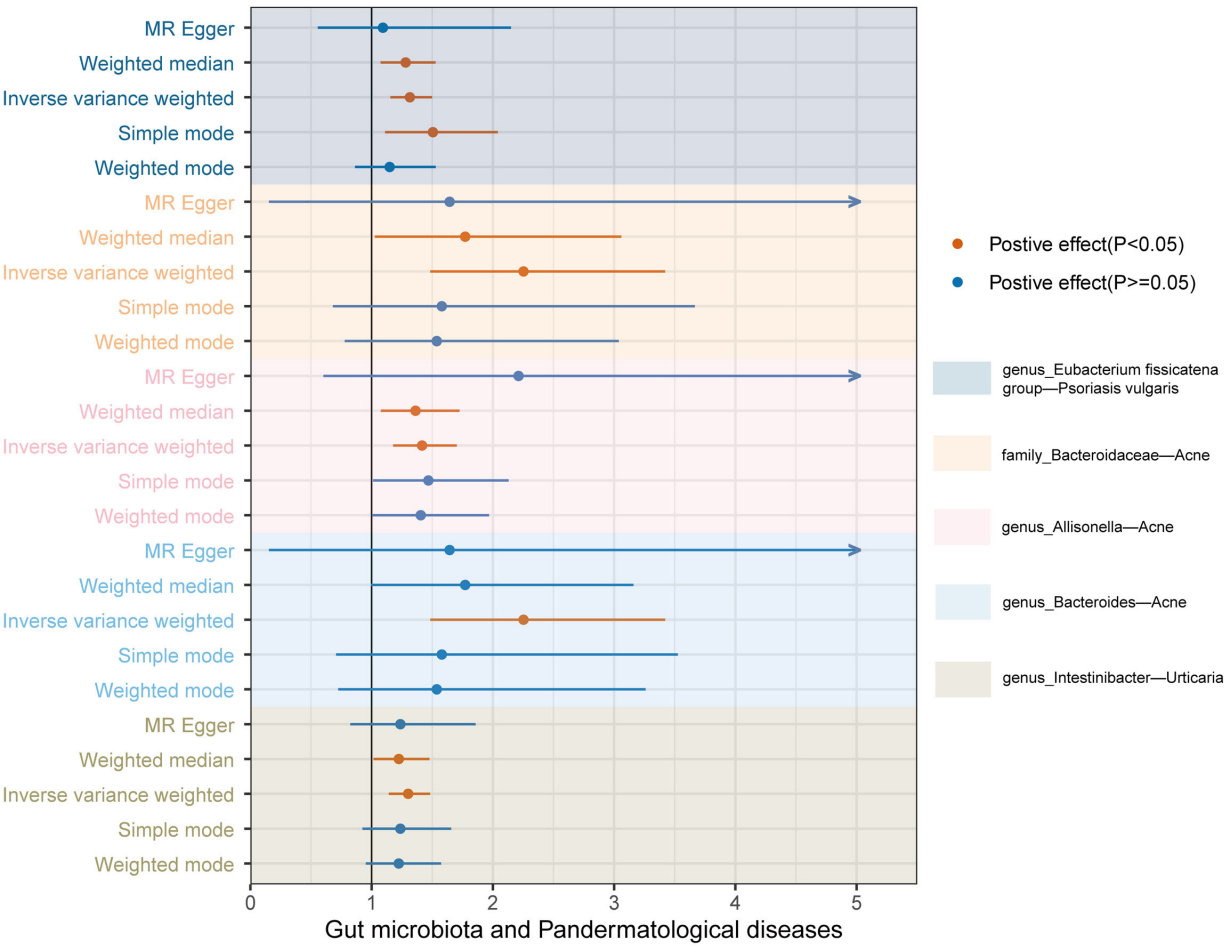
Forest plots of the MR results for the five identified significant causal effects. The horizontal coordinate represents the odds ratio value, dots depict the point estimate of odds ratio, and horizontal bars depict the 95% confidence interval. Orange represents significant results, whereas blue represents non-significant results. The arrow in the figure indicates that the upper limit of the 95% confidence interval of the odds ratio value under the method exceeds the upper limit of the horizontal coordinate, which is what we did for the aesthetics of the image.

**Figure 3:**
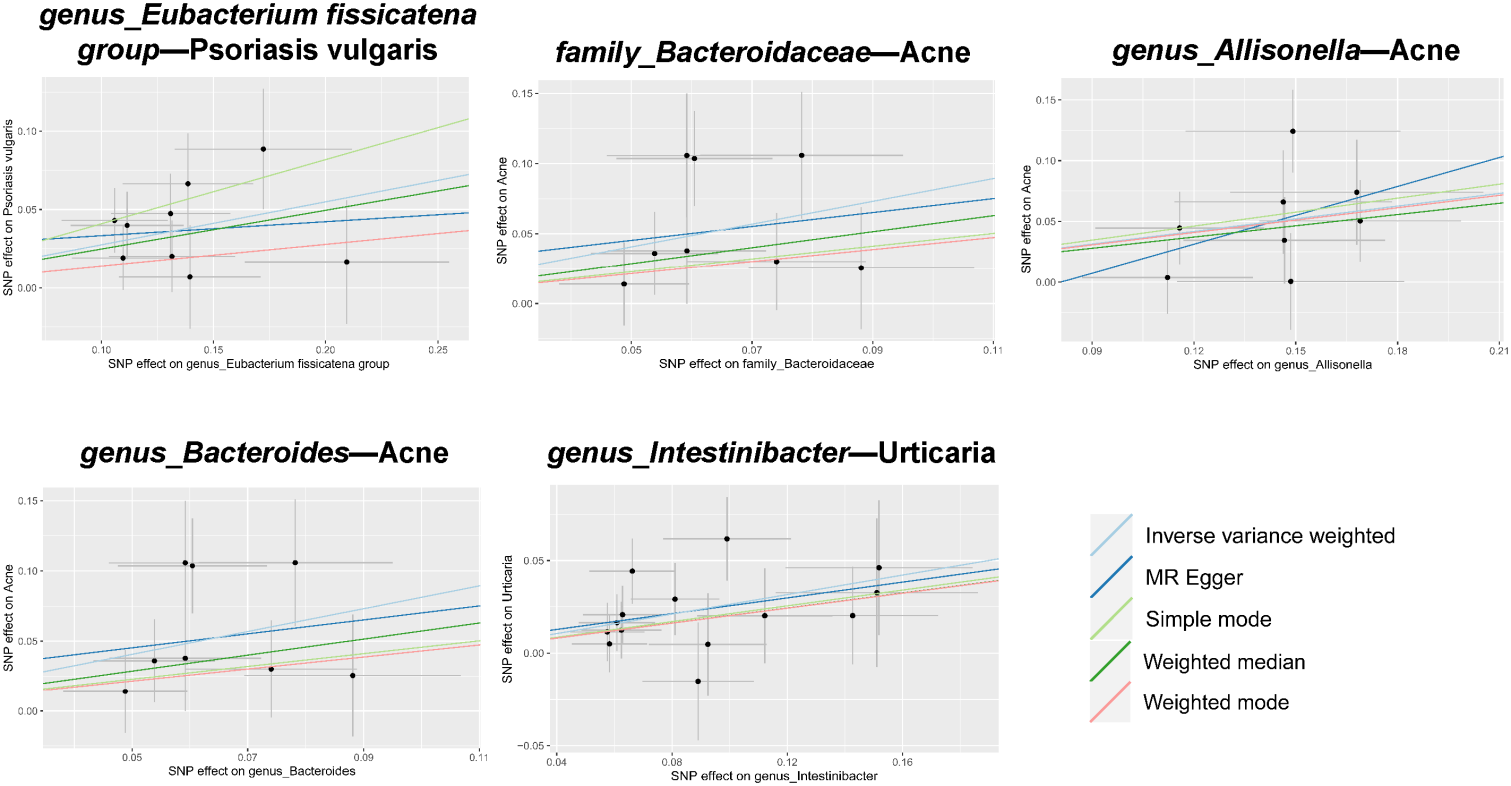
Scatter plots of estimates for significant associations between gut microbiota and dermatoses. Causal influence is represented by the slope value, which is equal to the b-value computed using the five methods. A positive slope indicates a risk factor for exposure.

In tandem with the IVW method, four robust methods, namely, MR–Egger, weighted median, weighted mode, and simple mode, were applied to evaluate the reliability of the causal estimates in our analysis. Partial methods generated similar and significant causal estimates while all methods exhibited concordant directional causal estimates (**Figure 2** and **Table 1**), i.e., the *genus Eubacterium_fissicatena_group* promoted the induction of psoriasis vulgaris (P_IVW_ = 3.66 × 10^−5^, P_simple mode_ = 2.99 × 10^−2^, P_weighted median_ = 5.90 × 10^−3^), *genus Intestinibacter* promoted the induction of urticaria (P_IVW_ = 7.67 × 10^−5^, P_weighted median_ = 3.41 × 10^−2^), *family Bacteroidaceae* (P_IVW_ = 1.37 × 10^−4^, P_weighted median_ = 4.01 × 10^−2^), and *genus Allisonella* (P_IVW_ = 2.16 × 10^−4^, P_weighted median_ = 1.04 × 10^−2^) promoted the induction of acne. The scatter plots of these significant associations are shown in **Figure 3**. It is noteworthy to highlight that the IVW method (OR = 1.22, p = 1.81 × 10^−4^, p_FDR_ = 2.15 × 10^−2^) suggests an increased risk of psoriasis associated with the *genus Eubacterium_fissicatena_group*, in stark contrast to the direction derived from MR-Egger (OR = 0.82). Considering this disparity, we opted to exclude this contentious causal association. In summary, we assert that the results of the IVW regression analysis remain steady and substantiated.

### Suggestive causal associations between gut microbiota and dermatological diseases

**Figure 4:**
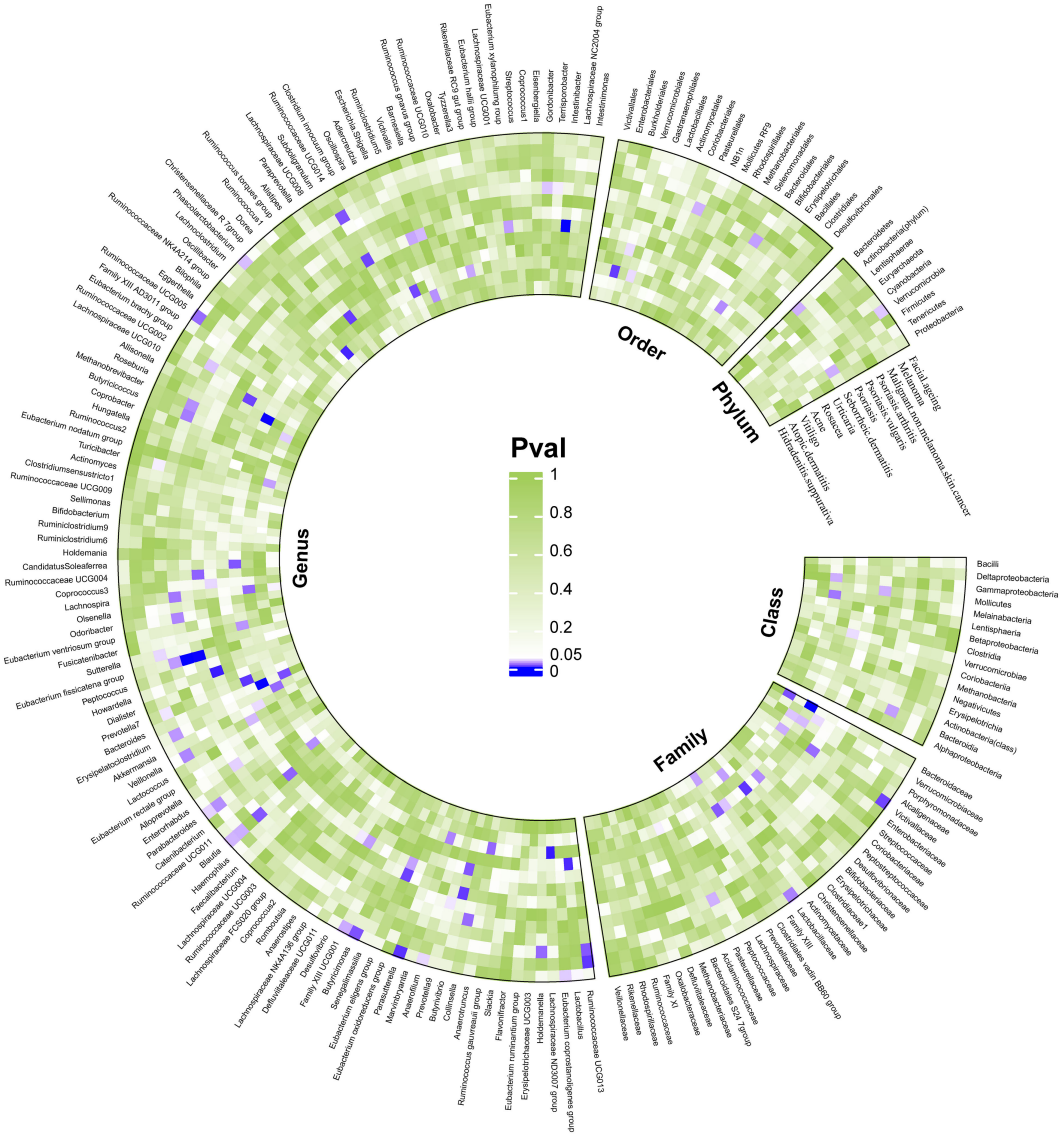
Suggestive causality of the gut microbiota in dermatoses derived from the inverse variance weighted method. Estimates with p <0.05 were shown in purple or blue, while estimates with p ≥0.05 were shown in white or green.

We considered causal relationships with p-values <0.05 but p_FDR_ ≥0.05 derived from the IVW method as potential or suggestive, while at the same time we discarded those associations in which horizontal pleiotropy was detected by MR-Egger intercept and MR-PRESSO even after pleiotropic SNP culling. In total, 94 suggestive relationships were identified (**Figure 4** and **Supplementary Tables 3, 4**).

#### Atopic dermatitis

Although no flora were found to have a significant association with AD, we still found eight taxa with a potential causal relationship: At the family level we were surprised to find that *family Bacteroidaceae* (OR = 1.36, p = 1.19 × 10^−2^) and *family Christensenellaceae* (OR = 0.81, p = 2.96 × 10^-2^) had inducing and protective effects, respectively; In addition, *genus Eubacterium_fissicatena_group* (OR = 1.12, p = 4.98 × 10^−2^), *genus Eubacterium_nodatum_group* (OR = 1.11, p = 2.98 × 10^−2^), *genus Bacteroides* (OR = 1.36, p = 1.19 × 10^−2^), *genus Christensenellaceae R_7 group* (OR = 0.71, p = 3.84 × 10^−3^), *genus Roseburia* (OR = 1.22, p = 3.96 × 10^−2^), *genus Ruminiclostridium_5* (OR = 0.79, p = 2.63 × 10^−2^) have been observed to have a suggestive causal associations.

#### Vitiligo

We identified 15 taxa potentially associated with vitiligo, including two classes, two orders, three families, and eight genuses. Among them, we found *class Gammaproteobacteria* (OR = 0.67, p = 3.29 × 10^−2^), *class Melainabacteria* (OR = 0.78, p = 2.06 × 10^−2^), *order Gastranaerophilales* (OR = 0.81, p = 4.24 × 10^−2^), *family Bacteroidales S24-7group* (OR = 0.73, p = 4.67 × 10^−2^), *family Porphyromonadaceae* (OR = 0.55, p = 3.78 × 10^−2^), *family Victivallaceae* (OR = 0.83, p = 3.25 × 10^−2^), *genus Anaerotruncus* (OR = 0.66, p = 1.72 × 10^−2^), *genus Erysipelatoclostridium* (OR = 0.77, p = 1.76 × 10^−2^), *genus Lachnospiraceae ND3007group* (OR = 0.38, p = 2.47 × 10^−3^), *genus Marvinbryantia* (OR = 0.72, p = 2.33 × 10^−2^), *genus Oxalobacter* (OR = 0.82, p = 3.12 × 10^−2^) had a potential protective effect, while *order Burkholderiales* (OR = 1.54, p = 7.94 × 10^−3^), *genus Lachnospira* (OR = 1.79, p = 1.71 × 10^−2^), *genus Catenibacterium* (OR = 1.28, p = 2.56 × 10^−2^), *genus Adlercreutzia* (OR = 1.43, p = 1.02 × 10^−2^) appear to be associated with an increased risk of vitiligo.

#### Acne

It seems that, as expected, we found that increased abundance of the *order Bifidobacteriales* and *family Bifidobacteriaceae* (OR = 0.69, p = 2.52 × 10^−2^) was potentially associated with a reduced risk of acne development. Furthermore, *family Lactobacillaceae* (OR = 0.78, p = 3.77 × 10^−2^), *genus Ruminococcust_orques_group* (OR = 0.53, p = 8.42 × 10^−3^), *genus CandidatusSoleaferrea* (OR = 0.75, p = 1.31 × 10^−2^), *genus Fusicatenibacter* (OR = 0.71, p = 2.71 × 10^−2^), *genus Lactobacillus* (OR = 0.72, p = 4.55 × 10^−3^) were also found to have a potential protective effect against acne. While *family Clostridiaceae 1* (OR = 1.67, p = 7.06 × 10^−3^), *family FamilyXIII* (OR = 1.73, p = 1.44 × 10^−3^), *family Porphyromonadaceae* (OR = 1.57, p = 3.56 × 10^−2^) that are potentially associated with an increased risk of developing acne.

#### Rosacea

After MR-Egger intercept and MR-PRESSO analysis we discarded four suggestive causal associations, but even then, we still found that *genus Butyrivibrio* (OR = 0.83, p = 1.36 × 10^−2^) and *genus Prevotella7* (OR = 0.78, p = 6.95 × 10^−3^) were associated with a potential reduced risk of developing rosacea.

#### Urticaria

We found that *family Victivallaceae* (OR = 1.10, p = 1.84 × 10^−2^), *genus Coprococcus3* (OR = 1.24, p = 4.16 × 10^−2^), and *genus DefluviitaleaceaeUCG011* (OR = 1.19, p = 2.92 × 10^−2^)may increase the risk of urticaria, while increased abundance of *genus Eubacterium_eligens_group* (OR = 0.75, p = 2.99 × 10^−2^), *genus Eubacterium_xylanophilum_group* (OR = 0.84, p = 2.89 × 10^−2^), *genus Lachnospiraceae NK4A136group* (OR = 0.88, p = 4.55 × 10^−2^), *genus LachnospiraceaeUCG010* (OR = 0.80, p = 9.62 × 10^−3^), *genus RuminococcaceaeUCG011* (OR = 0.89, p = 1.62 × 10^−2^), and *genus Veillonella* (OR = 0.80, p = 3.50 × 10^−2^)may be associated with a reduced risk of urticaria.

#### Seborrheic dermatitis

We found a potential causal association between *phylum Tenericutes*, *class Mollicutes* (OR = 1.34, p = 3.30 × 10^−2^), and an increased risk of seborrheic dermatitis, and similar effects were observed in *genus RuminococcaceaeUCG014* (OR = 1.47, p = 9.90 × 10^−3^) and *Victivallis* (OR = 1.24, p = 3.51 × 10^−2^). In contrast, *genus Eubacterium_eligens_group* (OR = 0.53, p = 9.57 × 10^−2^), *genus Butyrivibrio* (OR = 0.82, p = 7.91 × 10^−3^), *genus Howardella* (OR = 0.77, p = 2.68 × 10^−3^), *genus Ruminiclostridium5* (OR = 0.67, p = 3.08 × 10^−2^), and *genus RuminococcaceaeUCG004* (OR = 0.73, p = 1.60 × 10^−2^)were found to have a potential protective effect against seborrheic dermatitis.

#### Psoriasis

Two taxa were found to have a potential protective effect, namely *phylum Bacteroidetes* (OR = 0.81, p = 3.30 × 10^−2^)and *genus Prevotella9* (OR = 0.87, p = 4.47 × 10^−2^).

#### Psoriasis vulgaris

We identified six taxa with potential protective effects, which were *genus Alloprevotella* (OR = 0.85, p = 4.03 × 10^−2^), *genus Collinsella* (OR = 0.73, p = 1.80 × 10^−2^), *genus Gordonibacter* (OR = 0.89, p = 3.76 × 10^−2^), *genus Lachnospira* (OR = 0.57, p = 2.08 × 10^−2^), *genus Odoribacter* (OR = 0.74, p = 2.37 × 10^−2^), and *genus Terrisporobacter* (OR = 0.79, p = 4.58 × 10^−2^).

#### Psoriatic arthritis

After excluding one finding with horizontal pleiotropy based on MR-Egger intercept, we still found eight taxa with potential associations with psoriatic arthritis, where *class Bacteroidia* (OR = 0.79, p = 4.58 × 10^−2^), *order Bacteroidales* (OR = 0.79, p = 4.58 × 10^−2^), *genus RuminococcaceaeUCG002* (OR = 0.79, p = 4.58 × 10^−2^) associated with a reduced risk of developing psoriatic arthritis; while *order Pasteurellales* (OR = 1.22, p = 3.33 × 10^−2^), *family Pasteurellaceae* (OR = 1.22, p = 3.33 × 10^−2^), *genus Eubacterium_fissicatena_group* (OR = 1.21, p = 2.83 × 10^−2^), *genus Blautia* (OR = 1.46, p = 1.36 × 10^−2^), *genus Methanobrevibacter* (OR = 1.27, p = 2.59 × 10^−2^) were associated with an increased risk of developing psoriatic arthritis.

#### Melanoma

We identified six taxa with suggestive causal associations. Four showed weak protective effects: *genus Blautia* (OR = 0.997, p = 3.28 × 10^−2^), *genus Erysipelatoclostridium* (OR = 0.998, p = 2.77 × 10^−2^), *genus Prevotella7* (OR = 0.999, p = 4.47 × 10^−2^), *genus RuminococcaceaeUCG013* (OR = 0.996, p = 7.11 × 10^−3^); Two showed weak evoked effects: *genus Parabacteroides* (OR = 1.003, p = 2.59 × 10^−2^) and *genus Veillonella* (OR = 1.003, p = 2.38 × 10^−2^).

#### Malignant non-melanoma skin cancer

In addition to *genus Turicibacter* (OR = 1.005, p = 4.64 × 10^−2^) which has the potential to increase the risk of skin cancer development, four other taxa including *genus Holdemanella* (OR = 0.995, p = 1.73 × 10^−2^), *genus RuminococcaceaeUCG013* (OR = 0.992, p = 1.38 × 10^−2^), *genus RuminococcaceaeUCG014* (OR = 0.993, p = 1.28 × 10^−2^) and *genus Sutterella* (OR = 0.994, p = 4.16 × 10^−2^) were potentially protective as well.

#### Facial aging

Although our MR analysis found a potential protective effect of *genus Butyricimonas* on facial aging, the MR-PRESSO results showed some level of pleiotropy (global test P-value = 2.92 × 10^−2^) and were not corrected after SNP culling, so we were skeptical of this result and excluded it. In addition, we observed that one, two, and nine taxa at the phylum, family, and genus levels, respectively, were potentially associated with facial aging. *Phylum Verrucomicrobia* (OR = 0.989, p = 3.83 × 10^−2^)was observed to have a potential protective effect; *family Victivallaceae* (OR = 1.007, p = 1.00 × 10^−2^) and *family Lactobacillaceae* (OR = 0.989, p = 2.25 × 10^−2^) had weak induced and protective effects, respectively; suggestive causal associations were also found between *genus Eubacterium_coprostanoligenes_group* (OR = 1.012, p = 3.92 × 10^−2^), *genus Anaerofilum* (OR = 0.993, p = 4.15 × 10^−2^), *genus Blautia* (OR = 1.011, p = 3.41 × 10^−2^), *genus FamilyXIIIUCG001* (OR = 0.987, p = 3.21 × 10^−2^), *genus Lactobacillus* (OR = 0.991, p = 4.83 × 10^−2^), *genus Parabacteroides* (OR = 0.984, p = 4.04 × 10^−2^), *genus Parasutterella* (OR = 1.014, p = 6.25 × 10^−3^), *genus Phascolarctobacterium* (OR = 0.989, p = 3.62 × 10^−2^), *genus RuminococcaceaeUCG005* (OR = 0.988, p = 1.79 × 10^−2^) and facial aging.

#### Hidradenitis suppurativa

MR analysis revealed that the genus Lachnospira (OR = 0.434, p = 4.73 × 10^−2^) constituted a risk factor, whereas the Family XIII (OR = 2.272, p = 3.01 × 10^−2^) was identified as a protective factor.

### Sensitivity analysis

**Figure 5:**
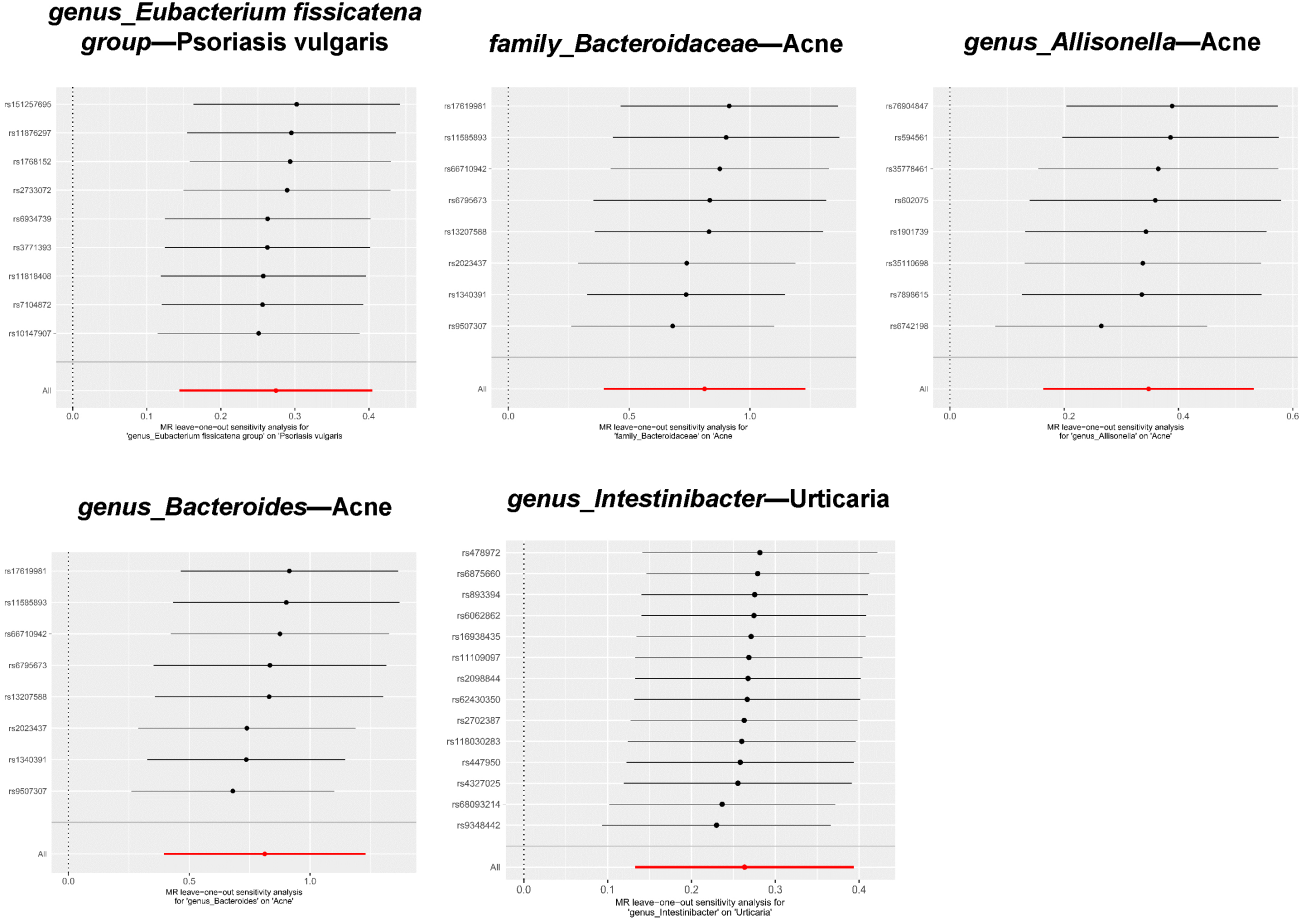
Leave-one-out plots for significant associations between gut microbiota and dermatoses.

Among the five significant causal associations, the robustness of our conclusions was underscored by the absence of detected heterogeneity or horizontal pleiotropy (**Table 1** ). Additionally, with respect to the 94 suggestive causal associations identified, the vast majority exhibited homogeneity according to the Cochrane Q test (**Supplementray Table 6**). Sporadic instances of heterogeneity were accommodated within the framework of the IVW random effects model. Notably, out of the extensive analysis performed, a mere six (5.66%) exhibited pleiotropic effects, as identified through the MR Egger intercept (**Supplementray Table 5**) and MR-PRESSO (**Supplementray Table 7**), which is detailed in the *Results* section. Ultimately, the leave-one-out test did not identify any biased SNPs (**Figure 5** and **Supplementray Table 8**).

### Reverse MR analysis

The inverse correlations that we found were suggestive and not significantly causal. Psoriasis vulgaris was linked to an increased relative abundance of the *genus Alloprevotella* (beta = 0.20, p = 2.63 × 10^−2^, p_FDR_ = 0.18); psoriatic arthritis was linked to an increased relative abundance of *class Bacteroidia* (beta = 0.042, p = 4.72 × 10^−2^, p_FDR_ = 0.13), *order Bacteroidales* (beta = 0.042, p = 4.72 × 10^−2^, p_FDR_ = 0.13), and *genus Ruminococcaceae UCG002* (beta = 0.046, p = 2.10 × 10^−2^, p_FDR_ = 0.13). We also found that vitiligo may lead to an increase in the abundance of the *order Burkholderiales* (beta = 0.022, p = 1.33 × 10^−2^, p_FDR_ = 0.20), while urticaria leads to the *family Victivallaceae* (beta = -0.26, p = 3.35 × 10^−2^, p_FDR_ = 0.34) decrease. Finally, there was a potential relationship between facial aging and increased abundance of the *family Lactobacillaceae* (beta = 0.63, p = 1.90 × 10^−2^, p_FDR_ = 0.19) and *genus Lactobacillus* (beta = 0.59, p = 2.87 × 10^−2^, p_FDR_ = 0.19). The results of reverse MR analysis are presented in **Supplementray Tables 9, 10**. None of the above analyses revealed heterogeneity or pleiotropy (**Supplementary Tables 11–13**).

## Discussion

In the present study, we found 99 promising associations between the genetically predicted abundance of specific bacterial taxa and 13 dermatological diseases using large-scale GWAS summary data via bidirectional 2SMR analysis. After correction for multiple testing, we found that the enrichment of five taxa, namely *Eubacterium_fissicatena_group*, *Bacteroidaceae*, *Allisonella*, *Bacteroides* and *Intestinibacter*, was significantly associated with an increased risk of developing skin diseases.

The skin and intestines are intricate, dynamic immune and neuroendocrine organs that act as the body’s primary contact points for the external milieu. Both organs are imperative for upholding the equilibrium of physiological homeostasis (O’Neill et al., 20162; Coates et al., 2019). The gut is replete with microorganisms and it is estimated that it contains approximately 10^14^ microbial cells (Williams, 1973). The strong immunomodulatory potential of the gut microbiota, particularly in distal organs including the lungs, brain, and skin, has given rise to the gut–lung axis, gut–brain axis, and gut–skin axis as hot study fields (Chen et al., 2021; De Pessemier et al., 2021). Several studies have linked gastrointestinal health to skin homeostasis, and there is evidence of a bidirectional link between gut microbiota dysbiosis and skin homeostatic imbalance. Gut microbiota dysbiosis plays a specific role in the pathophysiological processes of several dermatologic diseases, involving the immune and neuroendocrine systems (Shah et al., 2013; Thrash et al., 2013; Salem et al., 2018). Disturbances in the gut microbiota can lead to increased epithelial permeability of the intestinal mucosa and activation of effector T cells, disrupting the balance between the gut microbiota and intestinal mucosa, whereas proinflammatory cytokines can further increase epithelial permeability and promote chronic systemic inflammation (Brown et al., 2019). The gut microbiota can produce noxious substances that are subsequently taken up by the bloodstream and circulate, harming faraway places such as the skin. For example, gut microbiota constituents, such as *C. difficile*, can metabolize aromatic amino acids to produce free phenols and p-cresols, which can enter the blood circulation and accumulate in the skin (Dawson et al., 2011). *In vitro* experiments have shown that p-cresol and phenol reduce keratin 10 expression in keratinocytes, thereby affecting epidermal differentiation and barrier function (Miyazaki et al., 2014). In addition, intestinal bacteria themselves may also enter the circulation through the damaged intestinal barrier and then reach the skin to cause disease; for example, some studies have discovered that the blood of patients with psoriasis contains DNA originating from intestinal bacteria (Ramírez-Boscá et al., 2015).

*Bacteroides* is a gram-negative anaerobic bacterium and a core member of *Bacteroidetes*, which has multiple roles as a beneficial microorganism, intestinal competitor, and an opportunistic pathogen. It metabolizes long-chain polysaccharides and oligosaccharides to provide nutrients to other gut microorganisms via a comprehensive system of glycoside hydrolases, polysaccharide lyases, sugar transporters, and carbohydrate-degrading enzymes (Zafar and Saier, 2021). Overpopulation of *Bacteroides* causes degradation of intestinal mucus, which can lead to disruption of the intestinal barrier and thus may allow translocation of pathogenic microorganisms across the gut (Desai et al., 2016). It also acts as an opportunistic pathogen that can cause infections by colonizing the intestinal tract (Michaudel and Sokol, 2020). In this study, we found that enrichment of the *family Bacteroidaceae* and *genus Bacteroides* was significantly associated with an increased risk of acne development, while we also found a positive correlation between AD and the *genus Bacteroides*. There have been studies revealing the possible mechanisms of *Bacteroides* in the pathogenesis of acne. *Bacteroides*, a gram-negative bacterium, is rarely found on the skin; however, Li et al. found that the abundance of *Bacteroides* was significantly elevated in the skin flora of patients with severe acne and suggested that the possible mechanisms include promotion of inflammation in acne, impairment of immune defenses, reduction of tissue repair, and inhibition of resident skin bacterial growth (Li et al., 2019). In addition, given the increased intestinal permeability caused by *Bacteroides* overgrowth, more gram-negative bacterial lipopolysaccharides (LPS) may enter systemic circulation and contribute to the development of inflammation and acne lesions through the action of Toll-like receptor (TLR) 2 and TLR4, whose expression is upregulated in acne (Juhlin and Michaëlsson, 1983; Terhorst et al., 2010; Maronese et al., 2023). Toxin-producing strains of *Bacteroides* have been shown to be associated with ulcerative colitis, toxin-mediated acute diarrhea, etc., because they have the most complex polysaccharide structure of any enterobacteria and the potent virulence factors, hemolysin/cytolysin, capable of lysing and killing host immune cells (Zamani et al., 2017; Valguarnera and Wardenburg, 2020; Zafar and Saier, 2021). We hypothesized that these properties are important in the mechanism by which the bacterium exacerbates acne, but the exact mechanism still needs to be elucidated. We know very little about *Bacteroidaceae*, except that *Bacteroides* is the most important member of the family; perhaps the positive effects on acne that we have found are the result of *Bacteroides* or other genera within the family may also contribute, but these conclusions require additional research and analysis. A correlation between *Bacteroides* and AD has also been reported, with two studies in 2021 reporting a higher abundance of *Bacteroides* in patients with AD (Su et al., 2021; Ye et al., 2021). The abundance of *Bacteroides* is influenced by pregnancy, feeding status, and antibiotic use early in life, and lower intestinal bacterial diversity in infancy and higher levels of *Bacteroides* are thought to be associated with an increased risk of atopic diseases (Wall et al., 2009; Lee et al., 2014). It has been found that higher levels of *Bacteroides* in allergic patients may lead to the persistent production of LPS, which induces an imbalance between the Th1 and Th2 immune responses through overactivation of TLRs, damage to the epidermal barrier, and itch-induced scratching, ultimately leading to the development of AD (Sipka and Bruckner, 2014; Behzadi et al., 2021; Fang et al., 2021). In addition, although we did not find a causal relationship between *Bacteroides* and psoriasis, we found that the *phylum Bacteroidetes* had a probable protective effect in patients with psoriasis, which is in line with the results of previous studies (Huang et al., 2019; Polak et al., 2021). Previous research has suggested that *Bacteroidetes* can produce acetate and propionate, both of which are anti-inflammatory, help maintain the epithelial barrier, protect against colitis, reduce oxidative stress, and regulate the balance between Th17/Treg lymphocytes (Myers et al., 2019), which may have a protective effect against psoriasis. There are currently conflicting findings on changes in the abundance of *Bacteroides* in the gut of patients with psoriasis; with one study (Tan et al., 2018) reported an increase in abundance and three studies (Codoñer et al., 2018; Hidalgo-Cantabrana et al., 2019; Dei-Cas et al., 2020) reported the opposite, a point of contradiction that deserves follow-up. In conclusion, *Bacteroides* is a key component of the gut microbiota and a double-edged sword that can provide nutrients to other flora through robust metabolism, generate short-chain fatty acids (SCFAs) that exert anti-inflammatory and immunomodulatory effects, and become pathogenic when their abundance and colonization status are changed. Skin may be a target organ for pathogenicity in an imbalanced state (Zafar and Saier, 2021).

In addition to *Bacteroides*, three other members of the flora that were strongly dermatologically associated attracted our attention. The *Eubacterium_fissicatena_group*, a specific group within *Eubacterium*, can metabolize and produce SCFAs, and *Eubacterium* is currently considered to play a beneficial role in human health, along with *Lactobacillus* and *Bifidobacterium* (Mukherjee et al., 2020). However, we made a groundbreaking discovery that the *genus Eubacterium_fissicatena_group* enrichment leads to a potentially increased risk of psoriasis, psoriasis vulgaris, and psoriatic arthritis in hosts, and its causal association with psoriasis vulgaris was more significantly correlated after a more stringent FDR correction. In fact, a previous Spearman correlation analysis revealed that the *Eubacterium_fissicatena_group* was positively associated with proinflammatory markers such as TNF-α, IL-6, and IL-8, negatively associated with anti-inflammatory markers such as IL-10, and associated with colonic inflammation in inflammatory bowel disease (IBD) (Liu et al., 2022). In addition, some researchers have found that *Eubacterium_fissicatena_group* is highly correlated with obesity and obesity-related metabolic disorders (Song et al., 2021). Both IBD and obesity have been shown to be associated with a high risk of developing psoriasis, and gut microbiota dysbiosis is thought to mediate the development of both condidtions (Maronese et al., 2021; Rogler et al., 2021; Barros et al., 2022). Therefore, we hypothesized that the *Eubacterium_fissicatena_group* may contribute to the pathogenesis of psoriasis by inducing colonic inflammation and metabolic disorders. However, details of this mechanism remain unclear. We also found a significant positive causal relationship between *genus Allisonella* and acne. To our knowledge, this is the first report of a relationship between this bacterium and acne, which utilizes histidine decarboxylation as its sole source of energy, and is a histamine-producing bacterium (Garner et al., 2002). Histamine can cause vasodilation and increase vascular permeability, as well as modulate immune cell activity and inflammatory factor release (Koh et al., 2002). We hypothesized that the bacterium may play a proinflammatory role in the development of acne-associated inflammation by releasing histamine, but this hypothesis needs to be confirmed by further studies. *Intestinibacter* belongs to the *Clostridiaceae* family in the phylum *Firmicutes* and is a genus of SCFA-producing bacteria (Dong and Yang, 2014). Despite growing evidence of significant differences in gut microbiota composition and metabolic function in patients with urticaria compared to healthy populations (Lu et al., 2019; Wang et al., 2021; Zhang et al., 2021), our study showed for the first time a significant correlation between increased abundance of the *genus Intestinibacter* and the risk of urticaria development, which was statistically significant even after FDR correction. There is limited research on this flora constituent, and studies have reported that the abundance of *Intestinibacter* is associated with type 2 diabetes mellitus (Neri-Rosario et al., 2023), Crohn’s disease (Forbes et al., 2018), prenatal depression (Fang et al., 2023), and osteoporosis (Akinsuyi and Roesch, 2023) and is also influenced by the HLA genotype (Forbes et al., 2018), but its definitive role remains unknown. Functional analysis of *Intestinibacter* has shown that it is able to degrade fucose, suggesting an indirect involvement in intestinal mucus degradation (Mueller et al., 2021), leading to a compromised intestinal barrier that allows microbes and toxins to infiltrate the body’s circulation and skin, triggering an immune response; however, whether there is a link between this activity and the development of urticaria remains to be investigated.

The gut microbiota has also been more broadly associated with dermatological diseases, and there are links between multiple taxa and multiple dermatological diseases. Although these associations are considered only suggestive, due to our application of rigorous statistical methods, they still provide some explanation for the role of the flora in the pathogenesis of dermatological diseases, at least qualitatively and with a clear causal direction. *Prevotella* is an SCFA-producing bacterium that has been found to enhance intestinal barrier function and reduce the levels of inflammatory indicators in the cecum (Neyrinck et al., 2011, 2012). Our study showed the protective effects of the *genus Prevotella7* against melanoma and rosacea and the likely protective effects of the *genus Prevotella9* against psoriasis. An analysis of the gut microbiota of 15 rosacea patients and 15 healthy individuals showed that *Prevotella* was more enriched in the intestines of healthy individuals than in patients (Moreno-Arrones et al., 2021). Given that *Prevotella* has been found to be more scarce in the gut of Parkinson’s disease patients and that rosacea and Parkinson’s disease are epidemiologically linked, further exploration of the role of this bacterium in the brain–gut–skin axis is warranted (Egeberg et al., 2016; Gerhardt and Mohajeri, 2018). Cutaneous melanoma is a highly malignant and metastatic tumor. The advent of immune checkpoint inhibitor (ICI) therapies has made it possible to harness the immune system to treat cancer. Inhibitory programmed cell death 1/programmed cell death ligand 1 or cytotoxic T-lymphocyte-associated protein 4 pathways allow malignant tumors to evade the immune system. By blocking these signaling pathways, ICI therapy allows the immune system to re-identify and kill tumors (Marincola et al., 2003; Gopalakrishnan et al., 2018). Peters et al. found that *Prevotella stercorea* was associated with longer survival during ICI treatment of metastatic melanoma (Peters et al., 2019), albeit with a different strain than the one we found, which was sufficient to focus our attention on the genus *Prevotella*. A point of contradiction occurs in the relationship between *Prevotella* and psoriasis, as a case–control study from Brazil showed an increased abundance of *Prevotella copri* in patients with psoriasis (Schade et al., 2022), whereas 16S rRNA sequencing by Shapiro et al. showed a decrease in the abundance of *Prevotella copri* in patients with psoriasis (Shapiro et al., 2019), which seems to support the latter finding. However, a study by Zhao et al. showed that after transplanting the fecal microbiota of psoriasis model mice with a severe skin phenotype to mildly symptomatic mice, the latter exhibited an exacerbation of psoriasis-like skin inflammation, including increased Th17 infiltration and differentiation, as well as an increase in *Prevotella* abundance in the colon (Zhao et al., 2023). In addition, they found that altered *Prevotella* abundance caused disturbances in fatty acid metabolism in the gut, such as an increase in oleic and stearic acid levels, both of which have been shown to exacerbate psoriasis-like skin inflammation by promoting the differentiation of Th17 cells and inducing IL-23 secretion by dendritic cells of monocyte origin (Zhao et al., 2023).

Despite accounting for less than 1% of the total number of human distal gut bacteria, *Lactobacillus* can have a profound impact on human health (Heeney et al., 2018). *Lactobacillus* can promote intestinal health by improving intestinal bacterial composition, protecting the intestinal mucosal barrier, and modulating the intestinal immune response, which in turn improves the health of the body (Ingrassia et al., 2005; Seth et al., 2008; Heeney et al., 2018). Unexpectedly, we identified a potential protective effect of the *genus Lactobacillus* and *family Lactobacillaceae* against acne and facial aging. Previous studies have shown a decreased abundance of *Lactobacillus* in patients with acne compared to healthy populations (Deng et al., 2018; Thompson et al., 2020), and many studies have confirmed the ameliorative effect of *Lactobacillus* probiotics on acne (Yu et al., 2020). Some *Lactobacillus* strains have been found to reduce sebaceous triglyceride levels, enhance skin hydration, upregulate the expression of the moisturizing factor ceramide, inhibit *Propionibacterium acnes* proliferation, ameliorate insulin resistance, normalize IGF-1 gene expression, and improve epidermal barrier function (Gueniche et al., 2010; Fabbrocini et al., 2016; Kim et al., 2021; Tsai et al., 2021). The relationship between the gut microbiota and aging has also been recognized in recent years, with the results of a Korean study showing that elderly individuals in long-lived villages had a higher abundance of *Lactobacillus* (Kim et al., 2019). *Lactobacillus* can interact with dermal fibroblasts in a photoprotective manner to exert an anti-skin aging effect and exerts an anti-inflammatory effect by regulating intestinal cell tight junctions and downregulating matrix metalloproteinase (MMP) expression (Lee et al., 2021). *Lactobacillus* can also prevent skin wrinkles from photoaging by inhibiting the activities of MMP and elastase (Lim et al., 2020). In addition, a recent study showed that nutritional supplements, including *Lactobacillus*, can increase telomere length in healthy middle-aged adults (Tsoukalas et al., 2019). *Ruminococcaceae* is a group of strictly anaerobic bacteria present in the colonic mucosal biofilm of healthy individuals (D. C. Rubin et al., 2014). *Ruminococcaceae* plays an important role in the maintenance of gut health through their ability to produce SCFAs. Our results showed that there is an association between different genera within *Ruminococcaceae* and different dermatological diseases. Among them, *genus RuminococcaceaeUCG002*, *genus RuminococcaceaeUCG013*, *genus RuminococcaceaeUCG014*, and *genus Ruminococcus torques group* likely had protective effects against psoriatic arthritis, melanoma, malignant non-melanoma skin cancer, and acne, respectively. Several previous studies have found that *Ruminococcaceae* may be a risk factor for patients with psoriasis, and that the abundance of *Ruminococcaceae* is increased in patients with psoriasis, leading to a decrease in the levels of medium-chain fatty acids, a potential protective factor in psoriasis (Chen et al., 2018; Hidalgo-Cantabrana et al., 2019). Gopalakrishnan et al. (2018) found that patients with a higher relative abundance of *Ruminococcaceae* in the gut microbiota had higher frequencies of effector CD4+ and CD8+ T cells and maintained cytokine capacity in the somatic circulation, which could be enhanced during ICI therapy by increased antigen presentation and improved effector T-cell function in the tumor microenvironment that enhances systemic and antitumor immune responses. Investigations into circulating T-cell counts as prognosticators of ICI efficacy in melanoma and non-malignant cutaneous neoplasms have been undertaken, and we posit that, in forthcoming inquiries, *Ruminococcaceae* may emerge as a prospective biomarker for predictive assessment (Zelin et al., 2022). Furthermore, *Ruminococcaceae* produces butyrate, which can effectively inhibit inflammation and is a potentially beneficial flora constituent for patients with acne (Deng et al., 2018; Sánchez-Pellicer et al., 2022). However, because our findings are only at the genus level, the specific mechanisms of the interaction between these genera and dermatological diseases remain to be determined.

The role of the flora is not generalized, as the same flora may play completely opposite roles in different dermatological diseases, such as *Victivallaceae*, which has a potential protective effect against vitiligo but is a risk factor for urticaria and facial aging, and *Blautia*, which is potentially protective against melanoma but is a risk factor for psoriatic arthritis and facial aging, suggesting that the flora may act through multiple mechanisms in the development of dermatological diseases. The same group of bacteria may play different roles in different gut microenvironments; however, these specific mechanisms are unclear and require further investigation. Based on the results of the inverse MR, we did not find a significant causal effect of dermatological diseases on the gut microbiota. Although the specific mechanisms are not yet clear, recent research suggests that the gut–skin axis represents a bidirectional relationship (Sinha et al., 2021; Mahmud et al., 2022). For example, it has been found that food allergies may be the result of skin barrier damage and that AD patients are allergic to peanuts due to exposure to peanut proteins in household dust, which ultimately leads to IgE-mediated mast cell expansion and degranulation in the gut (Bartnikas et al., 2013; Brough et al., 2015). Probiotics can manipulate the host microbiome and confer health benefits on patients. To date, many studies have explored the use of probiotics for the treatment of dermatological diseases. The probiotics commonly used today include the genera *Bifidobacterium*, *Enterococcus*, *Escherichia*, *Lactobacillus*, *Saccharomyces*, and *Streptococcus* (Al-Ghazzewi and Tester, 2014; Fijan, 2014). Probiotics can reduce intestinal and skin inflammation by increasing serum IL-10 levels and inducing expression by regulatory T cells, while decreasing IL-17 levels, can act as antioxidants, and can induce the expression of tumor-suppressor genes to fight tumor cells (Geuking et al., 2011; Levkovich et al., 2013; Zhong et al., 2014). SCFAs are a product of the fermentation of dietary fiber by many probiotics, and their components, such as acetate, propionate, and butyrate, can improve the function and integrity of the intestinal epithelial barrier by increasing the expression of the tight junction proteins claudin-1 and zonula occludens-1, inhibiting the proliferation, migration, and adherence of inflammatory cells, as well as the production of cytokines, such as IFN-γ, to ultimately suppress inflammation and immune response (Maslowski et al., 2009; Wang et al., 2012; Sun et al., 2017). These SCFAs are essential carbon and energy sources for colonic enterocytes (Wong et al., 2006), and their absence results in functional disorders of colonic mucosa (Vb and Tm, 2004). A meta-analysis of 1,070 children reported a significant reduction in AD score (SCORAD) in patients with AD who were orally administered the probiotics *Lactobacillus fermentum*, *Lactobacillus salivarius*, or mixed strains (Huang et al., 2017). Jung et al. described a probiotic treatment for acne. The probiotic group demonstrated equivalent efficacy to the minocycline group in a 12-week experiment using *Lactobacillus acidophilus*, *Lactobacillus delbrueckii bulgaricus*, and *Bifidobacterium bifidum* to treat acne, with the occurrence of skin lesions decreasing by 67% and fewer adverse effects (Jung et al., 2013). Currently, there is little research on the therapeutic benefits of probiotics, many of which focus on AD, acne, psoriasis, and melanoma. Additional fundamental research and clinical studies are needed to better understand the microbiome as a risk factor for several dermatological illnesses and as a target for a cure.

In recent years, the methods of MR analysis have evolved, and because they can effectively overcome the disadvantages of traditional statistical methods, which are susceptible to the influence of external factors, and because the genotypes appear before the phenotypes, the results are not biased by the interference of reverse causality, making the conclusions more reliable and rigorous (Burgess et al., 2015; Bowden et al., 2018). Our investigation delved into SNPs derived from GWAS meta-analyses with substantial sample sizes, showing robust correlations with the gut microbiota and manifestations across diverse dermatological disease databases. Five robust analytical methods and rigorous statistical corrections were used to derive quantitative relationships. Sensitivity analysis showed no pleiotropy or heterogeneity, suggesting that our results are statistically robust, and that the strongly related taxa and multiple dermatological disease-related taxa identified in this study could provide a basis for future work. Notably, there is some discrepancy between our results and those of previous studies, which may be due in part to differences in sample size, ethnic background, dietary habits, sex distribution, and age among the subjects in different studies. Our study has some limitations. First, because the study population was largely of European origin, this may have led to biased estimates and affected the generalizability of the conclusions. Second, although the gut microbiota GWAS is the largest to date, its sample size remains modest and the number of loci tested is relatively limited. Third, for conducting sensitivity analyses and horizontal pleiotropy testing, sufficient genetic variants were required as IVs, consequently rendering the SNPs utilized in the analyses incapable of meeting the conventional GWAS significance threshold (p < 5 × 10^^-8^), potentially increasing the risk of false positives. Fourth, because the lowest taxonomic level in the gut dataset was genus, we could not further explore the causal relationships between the gut microbiota and dermatological diseases at the species level. Fifth, although our analysis supported conclusions regarding the causal relationship between certain flora constituents and dermatological diseases, their roles in pathogenesis remain unclear. We believe that future research should apply an integrated approach that utilizes multiple genomics, metabolomics, model recruitment transfer experiments, and relevant clinical trials to deepen the understanding of the influence of gut microbiota on the pathogenesis of dermatological diseases in the context of complex interactions between genes and the environment over time. Larger clinical trials of oral probiotics can also be conducted to identify the most effective species-specific combinations, dosages, and treatment durations for specific dermatological diseases, as well as to assess their safety and long-term benefits.

## Conclusions

In summary, our MR analyses yielded compelling evidence for a comprehensive, albeit suggestive, linkage between gut microbiota and pan-dermatological diseases, suggesting that diverse taxa may exert either predisposing or protective influences in the pathogenesis of various dermatoses. Moreover, we found five distinct cohorts of causal relationships marked by significant correlations. Our investigation provides a pivotal cornerstone in understanding the gut–skin axis, underscoring the potential for future multi-omics inquiries to elucidate the intricate mechanisms governing these causal associations and to facilitate the advancement of microbiota-centric preventive and therapeutic modalities.

## Data availability statement

The original contributions presented in the study are included in the article/**Supplementary Material.** Further inquiries can be directed to the corresponding author.

## Ethics statement

The studies involving humans were approved by The Second Affiliated Hospital and Yuying Children’s Hospital of Wenzhou Medical University, Wenzhou. The studies were conducted in accordance with the local legislation and institutional requirements. Written informed consent for participation was not required from the participants or the participants’ legal guardians/next of kin in accordance with national legislation and institutional requirements.

## Author contributions

YW: Writing – review & editing, Writing – original draft. TY: Data curation, Formal analysis, Writing – review & editing. YL: Writing – review & editing, Supervision, Visualization. HG: Investigation, Writing – review & editing. BH: Writing – review & editing, Investigation. YG: Writing – review & editing, Supervision. JW: Methodology, Supervision, Writing – original draft, Writing – review & editing.
